# Retinal Microvascular Dysfunction Reflects Vascular and Alzheimer's‐Related Pathology in Dementia With Lewy Bodies

**DOI:** 10.1002/cns.70891

**Published:** 2026-04-21

**Authors:** Qiuling Tong, William Robert Kwapong, Xiaoqian Luan, Suqing Hu, Yihan Hu, Yimo Guo, Chenli Ji, Ming Yang, Zhen Wang

**Affiliations:** ^1^ Neurology Department The First Affiliated Hospital of Wenzhou Medical University Wenzhou, Ouhai China; ^2^ Neurology Department Xuanwu Hospital, Capital Medical University Beijing China; ^3^ Geriatrics Department The First Affiliated Hospital of Wenzhou Medical University Wenzhou, Ouhai China; ^4^ Wenzhou Medical University Wenzhou, Ouhai China; ^5^ Integrated Traditional Chinese and Western Clinical Medicine Zhejiang Chinese Medical University Hangzhou China; ^6^ Neurology Department The Quzhou Affiliated Hospital of Wenzhou Medical University, Quzhou People's Hospital Quzhou China

**Keywords:** amyloid‐*β*, APOE ε4, cerebral small vessel disease, dementia with lewy bodies, optical coherence tomography angiography, phosphorylated tau, plasma biomarkers, retinal microvasculature

## Abstract

**Background:**

Dementia with Lewy bodies (DLB) frequently coexists with cerebrovascular injury and Alzheimer's‐related pathology, yet accessible in vivo markers of these processes remain limited. The retinal microvasculature shares structural and physiological characteristics with cerebral small vessels and may provide a non‐invasive window into neurovascular and neurodegenerative pathology.

**Methods:**

In this cross‐sectional study, 32 individuals with DLB and 31 age‐matched cognitively unimpaired controls (CU) underwent swept‐source optical coherence tomography angiography (OCTA), brain MRI, and plasma biomarker assessment. Retinal vessel densities of the superficial vascular complex (SVC), deep vascular complex (DVC), and choriocapillaris (CC) were quantified. Plasma amyloid‐*β*, phosphorylated tau‐217 (p‐tau217), and glial fibrillary acidic protein were measured. Cerebral small vessel disease (SVD) burden and white matter hyperintensity (WMH) volumes were derived from MRI. Associations with cognition and mediation by WMH burden were evaluated using generalized estimating equations and bootstrapped mediation analyses.

**Results:**

Compared with CU, individuals with DLB exhibited significantly reduced SVC, DVC, and CC vessel densities (all *p* < 0.001). Lower retinal vessel densities were associated with higher plasma amyloid burden and elevated p‐tau217, as well as greater SVD burden and periventricular WMH volume. APOE ε4 carriers demonstrated more pronounced retinal microvascular impairment, higher WMH burden, and elevated p‐tau217 levels than non‐carriers. Reduced SVC density was associated with worse global cognition, and this relationship was partially mediated by periventricular WMH volume.

**Conclusions:**

Retinal microvascular impairment measured by OCTA is closely linked to Alzheimer's‐related plasma biomarkers, SVD, and cognitive decline in DLB. These findings support retinal OCTA as a scalable, non‐invasive biomarker reflecting convergent neurodegenerative and vascular pathology in DLB.

## Graphical abstract

Retinal microvascular impairment is associated with elevated plasma amyloid and phosphorylated tau‐217, increased cerebral small vessel disease burden, and greater periventricular WMH volume. Reduced retinal perfusion is linked to worse cognitive performance, partially mediated by white matter hyperintensities.

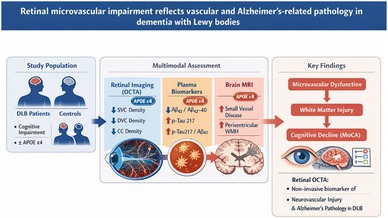

## 1 Background

Dementia with Lewy bodies (DLB) is the second most common cause of degenerative dementia in older adults after Alzheimer's disease (AD) and is characterized by progressive cognitive decline, fluctuating attention, visual hallucinations, Parkinsonism, and rapid eye movement sleep behavior disorder (RBD) [1]. The underlying neuropathological substrate includes α‐synuclein aggregation, often co‐occurring with amyloid‐*β* and tau pathology, contributing to substantial clinical and biological overlap with AD [2, 3, 4, 5]. Growing evidence suggests that DLB is further complicated by cerebrovascular injury, including small vessel disease and white matter hyperintensities, which may modify the clinical phenotype and accelerate cognitive decline [6, 7]. However, in vivo assessment of vascular contributions to DLB remains limited.

The retina provides a unique, non‐invasive window into the central nervous system structure and function. Retinal neurons share embryologic origin, microvascular architecture, and blood‐tissue barrier characteristics with the brain [8]. Optical coherence tomography angiography (OCTA) allows high‐resolution visualization of retinal capillary networks and has been increasingly applied in neurodegenerative research [9, 10]. Prior work has demonstrated reduced retinal vascular density in AD and Parkinson's disease (PD), supporting the hypothesis that neurodegeneration is accompanied by microvascular compromise [11, 12, 13]. Whether similar alterations are present in DLB and to what extent they relate to cerebral small vessel disease (SVD), plasma biomarkers of neurodegeneration, or cognition remains largely unknown.

Plasma biomarkers, including amyloid‐*β* isoforms and phosphorylated tau (such as p‐tau 217), now offer scalable approaches to quantifying core Alzheimer's‐related pathology in vivo [14, 15]. These markers may be elevated in DLB, particularly in individuals harboring mixed pathology or carrying the apolipoprotein E (APOE) ε4 allele [15]. Understanding how plasma biomarkers relate to retinal microvascular impairment may clarify intersecting pathogenic pathways linking amyloid deposition, tau phosphorylation, α‐synuclein aggregation, and vascular dysfunction.

In this context, OCTA‐derived microvascular metrics may serve as accessible indicators of neurodegenerative and vascular burden in DLB [16, 17]. However, their diagnostic performance, relationship to plasma biomarkers, association with SVD, and relevance to cognitive decline require further investigation. Addressing these gaps is particularly important for establishing multimodal biomarker frameworks that reflect disease biology across the brain and retina in DLB.

Accordingly, we evaluated retinal microvasculature using swept‐source OCTA in individuals with DLB and age‐matched cognitively unimpaired controls. We further examined the associations of OCTA‐derived perfusion metrics with plasma amyloid‐*β* and p‐tau 217 concentrations, white matter hyperintensity burden, and global cognition. We also explored the modifying effect of APOE ε4 carrier status. Our objective was to determine whether retinal vascular alterations reflect systemic and cerebral pathological changes in DLB and may provide clinically meaningful insight into disease mechanisms.

## 2 Methods

This study included participants evaluated between August 2025 and December 2025 at the First Affiliated Hospital of Wenzhou Medical University, China. Participants provided written informed consent at the time of enrollment, and the protocols of the study were approved by the Institutional Review Board of the First Affiliated Hospital of Wenzhou Medical University. The study followed the Strengthening the Reporting of Observational Studies in Epidemiology (STROBE) reporting guideline.

LBD patients included in the study met the 2020 Fourth Consensus Report of the DLB Consortium criteria [1]. All enrolled cases fulfilled criteria for probable DLB, based on ≥ 2 core clinical features or 1 core feature plus supportive biomarker evidence. Diagnoses were made by two board‐certified neurologists specializing in cognitive disorders (Zhen Wang). Other inclusion criteria were as follows: 1. Had full, detailed clinical information; 2. Normal appearance of optic nerve head and retina on ocular imaging; 3. Able to cooperate, undergo, and complete MR and OCTA imaging. Exclusion criteria were as follows: 1. Diagnosed with other forms of dementia or neurodegenerative disorders, such as frontotemporal dementia, or Parkinson's disease; 2. History or presence of ocular disease that could affect ophthalmic vascular metrics, such as diabetic retinopathy, optic neuropathy, or severe cataract; 3. History or presence of major psychiatric disorders, including schizophrenia, bipolar disorder, major depressive disorder with psychotic features, or other severe psychiatric illness that could confound cognitive or neuroimaging assessments; 4. History of vascular disorders such as uncontrolled hypertension and diabetes mellitus; 5. History of cancer; 6. History of substance abuse. Additional exclusion criteria included a diagnosis of epilepsy, epileptiform abnormalities on EEG, antidepressant‐triggered RBD, and RBD mimics such as sleepwalking, night terrors, or untreated obstructive sleep apnea.

The diagnosis of Rapid Eye Movement Sleep Behavior Disorder (RBD) was confirmed by video‐polysomnography according to the International Classification of Sleep Disorders (Third Edition) [18].

Age‐matched controls (CU) were enrolled from community‐dwelling citizens through advertisements from residents who attended our hospital for a health examination. Individuals who denied a history of neurologic disorder and attended our hospital for routine examination and did not show any abnormality on MR imaging were enrolled in our study and also were not impaired in any of the cognitive domains tested. Lifestyle, cerebrovascular risk factors, and clinical information were recorded for all controls. Exclusion criteria were similar to those of DLB.

Demographic data such as sex, age, education, and vascular risk factors (hypertension and diabetes mellitus) were recorded. All participants underwent Mini‐Mental State Examination (MMSE) and Montreal Cognitive Assessment (MoCA) as part of their cognitive assessment; these are brief screening dementia tools with a total score of 30, and a higher score indicates better cognition.

### 2.1 MR Imaging

A standardized protocol was conducted using a 3.0 T MRI system (SIGNA MR, GE Healthcare, WI, USA) in all participants. A full scanning sequence including the 3D T1‐weighted image, axial T2‐weighted image, axial T2 FLAIR image, and diffusion tensor imaging (DTI), and imaging parameters were the same as those we previously reported [12, 19]. In this study, the high‐resolution T1‐weighted image were acquired using a magnetization‐prepared rapid gradient‐echo (MPRAGE) sequence with the following parameters: echo time (TE) = 2.19 ms, repetition time (TR) = 2000 ms, inversion time = 900 ms, field of view (FOV) = 240 mm × 240 mm, matrix size = 256 × 256, flip angle = 8°, and voxel size = 0.9 mm × 0.9375 mm × 0.9375 mm.

### 2.2 Segmentation and Quantification of White Matter Hyperintensity Volume

The quantification of white matter hyperintensities (WMHs) was performed through automated segmentation techniques. Axial T2 fluid‐attenuated inversion recovery (FLAIR) imaging data were analyzed using the FreeSurfer WMH‐SynthSeg algorithm for its consistent performance even in low‐resolution MRI scans [20]. The results were further divided into periventricular WMHs (PWMHs) and deep WMHs (DWMHs), with the classification based on whether any voxel within a WMH cluster was located within 3 mm of the lateral ventricles [21]. PWMHs were defined as lesions located within 3 mm from the lateral ventricular wall and extending up to 10 mm from the lateral ventricle into the white matter. Confluent WMHs were defined as lesions within 3 mm of the lateral ventricular wall and extending more than 10 mm from the lateral ventricles into the white matter. DWMHs were defined as lesions separated from the margins of the lateral ventricles; the distance between deep WMHs and the ventricular wall was at least 3 mm. Periventricular and confluent WMHs were further analyzed as one group because differentiation between these two subtypes would affect the WMH shape calculations. To ensure precision, all segmentations underwent expert review by a neurologist (Z.W.) to minimize classification inaccuracies. For cross‐subject comparability, WMH volumes were normalized as a percentage of total intracranial volume (%ICV), thereby accounting for interindividual differences in brain size. Total intracranial volume was determined by summing the volumetric contributions of gray matter, white matter, cerebrospinal fluid, and WMH.

### 2.3 Plasma Analyses

Venous blood samples were obtained during morning hours; however, participants were not required to fast prior to collection. Blood was drawn into six K2‐EDTA anticoagulant tubes and delivered to the laboratory within 3 h for processing. Samples were centrifuged at 2000 × g at 4°C for 10 min. The resulting plasma was carefully separated, aliquoted into 1.5 mL polypropylene cryovials (1 mL per aliquot), and stored at −80°C until analysis. Plasma collection occurred within 1 month of the corresponding neuroimaging, retinal imaging, and clinical data assessments. All biochemical analyses were conducted at Nanjing Vazyme Biotech Co. Ltd., China, as previously reported [19, 22].

On the day of measurement, plasma aliquots were thawed at room temperature. To prevent variability related to repeated freeze–thaw cycles, only previously unthawed aliquots were used. Concentrations of amyloid‐*β*42 (A*β*42), amyloid‐*β*40 (A*β*40), and phosphorylated tau‐217 (p‐tau217) were quantified from 0.5 mL plasma samples using a fully automated chemiluminescent immunoassay system (Elf S240, Vazyme, Nanjing, China). The corresponding commercial assay kits (Vazyme *β*‐Amyloid 1–42, Vazyme pTau217 Plasma, and Vazyme NfL Plasma) were applied according to the manufacturer's standardized operating procedures. Following thawing, samples were vortex‐mixed and centrifuged to remove potential fibrin debris prior to assay loading [19].

### 2.4 SS‐OCTA Imaging

Retinal microvasculature was acquired by a swept‐source optical coherence tomography angiography system (SS‐OCTA, VG 200; SVision Imaging, Henan, China; version 3.1.352) between 8 am and 4 pm. The SS‐OCTA contained a swept‐source light source with a central wavelength of 1050 nm and a scan rate of 200,000 A‐scans per second, as previously reported [11, 23, 24, 25, 26, 27]. The tool was also equipped with an eye‐tracking utility based on an integrated confocal scanning laser ophthalmoscope to reduce and eliminate eye‐motion artifacts. The axial resolution, lateral, and scan depth were 5 μm, 13 μm, and 3 mm, respectively. Imaging was done in both left and right eyes without pupil dilation.

**FIGURE 1:**
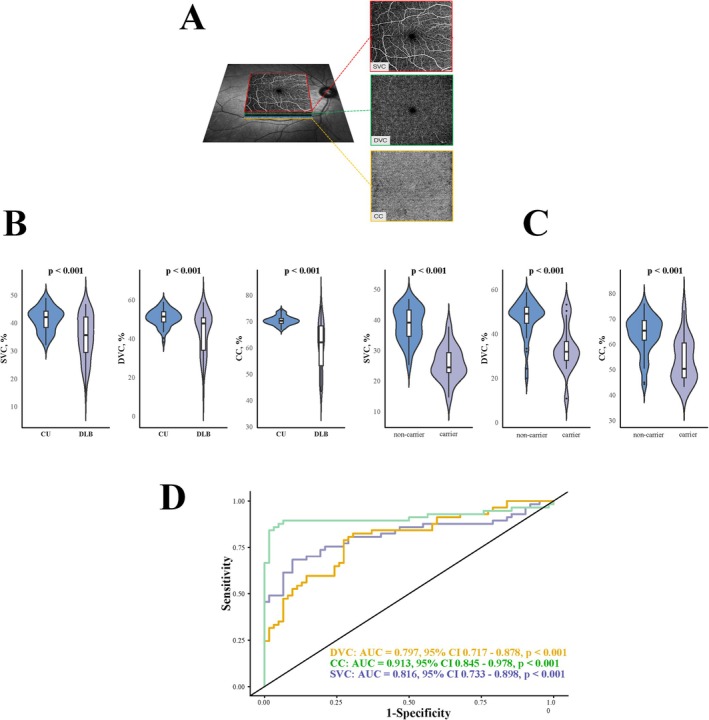
OCTA‐derived measures are sensitive to microvascular damage in Dementia with Lewy Bodies (DLB). (A) Segmentation of superficial vascular complex (SVC), deep vascular complex (DVC), and choriocapillaris (CC). (B) Comparison of OCTA‐derived measures between DLB and cognitively unimpaired controls (CU); DLB exhibited lower microvascular densities compared to CU. (C) Comparison of OCTA‐derived measures between DLB with APOE ε4 carriers and non‐carriers; Carriers showed significantly lower microvascular densities compared to non‐carriers. (D) ROC analysis of OCTA‐derived measures to differentiate DLB from CU; CC showed the highest area under the curve to differentiate DLB from CU.

OCTA fundus images were obtained with a raster scan protocol of 512 horizontal B‐scans that covered an area of 6 × 6 mm^2^ centered on the fovea. The B‐scans, which contained 512 A‐scans each, were repeated 8 times and averaged. Angiograms with signal quality of 8 and above were selected for further analysis. The en face angiograms of the superficial vascular complex (SVC) and deep vascular complex (DVC) of the retina were generated by automatic segmentation to evaluate retinal perfusions. The segmentation between the SVC and DVC was set in the inner two‐thirds and outer one‐third interface of the ganglion cell‐inner plexiform layer (GCIPL), as shown in Figure 1. The choriocapillaris (CC) was defined as a slab from the basal border of the retinal pigment epithelium‐Bruch's membrane complex to 20 μm below it. Vascular density (%) derived from the OCTA tool was used to evaluate the retinal microvasculature. The reproducibility of the OCTA metrics used in our study has been well documented in a previous study [28]. Angiograms used for processing followed the OSCAR‐IB quality criteria [29] and the APOSTEL recommendation [30].

All angiograms were assessed by a trained, certified grader masked to participant characteristics (X.L.). Angiograms with a signal quality (SQ) of ≥ 8 were considered eligible for further analysis. CFP and angiograms with motion artifacts, such as irregular microvascular patterns or blurred segmentation, were excluded from further analysis. Participants with retinopathy, such as age‐related macular degeneration, severe cataracts, glaucoma, optic neuritis, and hemorrhages, were excluded. If a participant presented with any of these disorders in one eye, the other eye was used; if both eyes had the aforementioned disorders, the participant was excluded from the study.

### 2.5 Statistical Analyses

The normality of the data was determined by visual inspection of the distribution and the Kolmogorov–Smirnov test. Consecutive variables with normal distribution were expressed as mean ± standard deviation (SD), and skewed distribution was expressed as median and interquartile range. Categorical variables are presented as frequencies and percentages (%). The Fisher's exact test, *t*‐test, or Kruskal–Wallis tests were used to compare clinical characteristics between DLB and CU.

A multiple linear regression model with a generalized estimating equation (GEE) was used to compare OCTA‐derived measures between CU vs. DLB and APOE ε4 carriers vs. non‐carriers in DLB while adjusting for risk factors. The covariates were set as age, sex, vascular risk factors (hypertension and diabetes mellitus), educational years, and the working correlation matrix was set as exchangeable, considering the correlation of the 2 eyes. Correction for multiple comparisons (3 OCTA metrics) was performed using the Bonferroni method with a significance level set at 0.05/3 ~ 0.0167.

The area under the receiver operating characteristic (AUROC) was calculated to determine the diagnostic capability of the OCTA metrics in DLB compared to CU; an AUROC of 1.0 represents perfect discrimination, while 0.5 indicates random discrimination.

GEE was also used to assess the association between OCTA‐derived measures with plasma biomarkers and neuroimaging metrics (SVD burden and WMH volumes) in DLB while adjusting for risk factors (age, sex, hypertension, diabetes mellitus) and intereye dependencies. Analyses were also conducted separately for the left and right eyes using general linear models to ensure the transparency of the results. A multiple linear regression model with GEE was also used to explore the association between OCTA‐derived measures and global cognitive scores in DLB while adjusting for risk factors. Results were reported as regression coefficients [*β*, per standard deviation (SD) increase/decrease] with corresponding 95% CIs.

Mediation analysis was performed to investigate whether the association between OCTA‐derived measures and global cognitive performance was mediated by neuroimaging measures (SVD burden and WMH volumes) in DLB. Non‐parametric bootstrapping (B = 5000) was used to compute 95% confidence intervals (95% CIs) for the total effect, mediation effects, and direct effects. For mediation analysis, age, sex, hypertension, diabetes mellitus, and educational years were adjusted.

Statistical analysis and plotting were conducted using *R* version 4.4.4 (gee package for GEE, ggplot, and dplyr). A *p* < 0.05 was considered significant.

## 3 Results

We initially enrolled 70 participants (37 DLB and 33 CU) in our study. After screening and imaging evaluation, we excluded 5 DLB and 2 CU due to poor neuroimages and uncooperativeness during assessment. Our final data analyses included 32 DLB and 31 CU. Demographics and vascular risk factors were comparable between the two groups, as shown in Table 1. Importantly, DLB patients showed significantly lower global cognitive scores compared to CU (all *p* < 0.001). Of the 32 DLB enrolled in our study, 17 (53.13%) had RBD.

**TABLE 1:** Demographic and clinical characteristics of our study participants.

	CU *N* = 31	DLB *N* = 32	
*Demographics*			
Eyes, *n*	64	57	
Age, years	72.07 ± 3.71	72.13 ± 9.29	0.973
Males, *n* (%)	11 (35.48%)	15 (46.88%)	0.508
Educational years	6 [6, 9]	6 [6, 9]	0.517
Disease duration		3 [2, 4]	
RBD		17 (53.13%)	
*Vascular risk factors*			
Hypertension, *n* (%)	11 (35.48%)	11 (34.38%)	1
Diabetes mellitus, *n* (%)	6 (19.35%)	5 (15.62%)	0.954
*Global cognition*			
MMSE	30 [29, 30]	16 [12, 21]	< 0.001
MoCA	29 [28, 30]	14 [8, 16]	< 0.001
*OCTA‐derived measures* *			
SVC, %	41.36 ± 4.25	34.85 ± 8.06	< 0.001
DVC, %	50.80 ± 4.33	43.35 ± 10.89	< 0.001
CC, %	70.35 ± 1.71	61.00 ± 9.02	< 0.001
*Neuroimaging measures*			
SVD burden	1 [1]	2 [1, 2]	< 0.001
TIV, mm^3^	1473.10 ± 97.84	1344.28 ± 76.09	< 0.001
PWMH, %ICV	0.23 ± 0.09	0.67 ± 0.35	< 0.001
DWMH, %ICV	0.02 ± 0.03	0.04 ± 0.04	0.043
TWMH, %ICV	0.25 ± 0.09	0.70 ± 0.35	< 0.001
*Plasma biomarkers*			
Aß42	8.58 ± 1.54	6.29 ± 1.67	< 0.001
Aß42/40	0.09 ± 0.01	0.04 ± 0.01	0.257
p‐tau 217	1.81 ± 0.96	11.90 ± 2.57	< 0.001
GFAP	70.63 ± 31.63	231.46 ± 88.97	< 0.001
p‐tau 217/Aß42	0.22 ± 0.13	2.01 ± 0.63	< 0.001

Abbreviations: CC: choriocapillaris; DVC: Deep vascular complex; DWMH: Deep white matter hyperintensity; GFAP: Glial fibrillary acidic protein; ICV: Intracranial volume; MMSE: Mini‐Mental State Examination; MoCA: Montreal Cognitive Assessment; PWMH: Periventricular white matter hyperintensity; RBD: Rapid eye movement sleep behavior disorder; SVC: Superficial vascular complex; SVD: Cerebral small vessel disease; TIV: Total intracranial volume; TWMH: Total white matter hyperintensity.

* Generalized estimating equation (GEE) was used to compare OCTA‐derived measures between CU and DLB while adjusting for age, sex, hypertension, diabetes mellitus, and educational years.

### 3.1 OCTA‐Derived Measures Are Sensitive to Microvascular Impairment in DLB

Compared to CU, DLB showed significantly reduced SVC (41.36% ± 4.25% vs. 34.85% ± 8.06%, *p* < 0.001), DVC (50.80% ± 4.33% vs. 43.35% ± 10.89%, *p* < 0.001), and CC (70.35% ± 1.71% vs. 61.00% ± 9.02%, *p* < 0.001) densities, as shown in Figure 1. We also found that CC showed the highest area under the curve (AUC: 0.913, Youden index = 0.830, Sensitivity = 89.5%, Specificity = 93.5%, 95% CI 0.845 to 0.978, *p* < 0.001; Figure 1), making it the most effective among all OCTA metrics for detecting microvascular changes in DLB and CU (Table S1).

Neuroimaging analyses showed that SVD burden and WMH volume were significantly elevated in DLB compared to CU. Further, we found that DLB showed significantly elevated plasma biomarkers compared to CU (all *p* < 0.001).

### 3.2 APOE ε4 Carrier Status Was Associated With Greater Retinal and Cerebral Vascular Impairment

Among our DLB, 9 tested positive for APOE ε4 while 23 tested negative. As shown in Table 2, baseline characteristics were comparable between the two groups. We found that carriers showed significantly reduced SVC (38.44% ± 5.65% vs. 25.64% ± 5.62%, *p* < 0.001), DVC (47.19% ± 8.31% vs. 33.51% ± 10.72%, *p* < 0.001), and CC (63.86% ± 7.54% vs. 53.66% ± 8.51%, *p* < 0.001) densities compared to non‐carriers. We also exhibited that carriers had significantly elevated SVD burden and WMH volumes compared to non‐carriers. Further, we found that plasma p‐tau 217 and p‐tau 217/A*β*42 were significantly elevated in carriers relative to non‐carriers (all *p* < 0.05).

**TABLE 2:** Comparison of OCTA‐derived measures between APOE ε4 carriers and non‐carriers in DLB.

	Non‐carriers *N* = 23	Carriers *N* = 9	*p*
Eyes, *n*	41	16	
Age, years	72.26 ± 9.51	76.89 ± 7.08	0.069
Males, *n* (%)	11 (47.83%)	4 (44.44%)	1
Educational years	6 [6, 9]	6 [6, 9]	0.965
Disease duration	3 [2, 4]	1 [1]	0.193
RBD	10	7	
*Vascular risk factors*			
Hypertension, *n* (%)	7 (30.43%)	4 (44.44%)	0.737
Diabetes mellitus, *n* (%)	2 (8.70%)	3 (33.33%)	0.236
Global cognition			
MMSE	18 [13, 21]	15 [9, 20]	0.702
MoCA	14 [10, 16]	9 [5, 14]	0.421
*OCTA‐derived measures*			
SVC, %	38.44 ± 5.65	25.64 ± 5.62	< 0.001
DVC, %	47.19 ± 8.31	33.51 ± 10.72	< 0.001
CC, %	63.86 ± 7.54	53.66 ± 8.51	< 0.001
*Neuroimaging measures*			
SVD burden	2 [1, 2]	2 [2, 3]	0.012
TIV, mm^3^	1340.57 ± 81.44	1353.75 ± 63.72	0.007
PWMH, %ICV	0.52 ± 0.22	1.04 ± 0.35	< 0.001
DWMH, %ICV	0.03 ± 0.03	0.06 ± 0.06	0.113
TWMH, %ICV	0.56 ± 0.22	1.07 ± 0.35	< 0.001
*Plasma biomarkers*			
Aß42	6.73 ± 1.84	5.44 ± 0.85	0.058
Aß42/40	0.09 ± 0.01	0.04 ± 0.01	0.257
p‐tau 217	11.21 ± 2.56	13.22 ± 2.13	0.045
GFAP	217.35 ± 71.54	258.13 ± 115.239	0.060
p‐tau 217/Aß42	1.77 ± 0.60	2.46 ± 0.40	0.006

Abbreviations: CC: choriocapillaris; DVC: Deep vascular complex; DWMH: Deep white matter hyperintensity; GFAP: Glial fibrillary acidic protein; ICV: Intracranial volume; MMSE: Mini‐Mental State Examination; MoCA: Montreal Cognitive Assessment; PWMH: Periventricular white matter hyperintensity; RBD: Rapid eye movement sleep behavior disorder; SVC: Superficial vascular complex; SVD: Cerebral small vessel disease; TIV: Total intracranial volume; TWMH: Total white matter hyperintensity.

* Generalized estimating equation (GEE) was used to compare OCTA‐derived measures between carriers and non‐carriers while adjusting for age, sex, hypertension, diabetes mellitus, and educational years.

### 3.3 OCTA‐Derived Measures Are Associated With Amyloid Burden and p‐Tau 217 in DLB

We found that elevated plasma amyloid burden in DLB was associated with reduced SVC and DVC densities (all *p* < 0.05). We also found that elevated plasma p‐tau 217 was associated with reduced SVC (ß = −2.315, *p* = 0.016) and CC (ß = −2.573, *p* = 0.034) perfusions in DLB. Importantly, we found that elevated plasma p‐tau 217/A*β*42 was associated with reduced SVC, DVC, and CC densities in DLB (all *p* < 0.05) as shown in Table 3.

**TABLE 3:** Association between OCTA‐derived measures, plasma biomarkers, and neuroimaging measures in DLB via generalized estimating equation.

SVC	*ß* (95% CI)	*p*	DVC	*ß* (95% CI)	*p*	CC	*ß* (95% CI)	*p*
*Plasma markers*								
Aß42	2.660 (0.758–4.562)	**0.010**	Aß42	2.907 (0.137–5.677)	**0.046**	Aß42	1.610 (−0.925–4.145)	0.221
Aß42/40	3.748 (1.987–5.510)	**< 0.001**	Aß42/40	2.923 (0.100–5.747)	**0.049**	Aß42/40	2.285 (−0.248–4.819)	0.084
p‐tau 217	−2.315 (−4.123–0.507)	**0.016**	p‐tau 217	−2.062 (−4.720–0.597)	0.136	p‐tau 217	−2.573 (−4.863–0.282)	**0.034**
GFAP	−1.154 (−3.233–0.925)	0.283	GFAP	−0.833 (−3.786–2.119)	0.583	GFAP	−2.649 (−5.147–0.151)	**0.044**
p‐tau 217/Aß42	−3.748 (−5.434–2.061)	**< 0.001**	p‐tau 217/Aß42	−4.056 (−6.645–1.467)	**0.003**	p‐tau 217/Aß42	−3.207 (−5.559–0.855)	**0.011**
*MRI markers*								
SVD burden	−3.574 (−5.623–1.526)	**0.001**	SVD burden	−3.851 (−6.608–1.094)	**0.009**	SVD burden	−3.079 (−5.519–0.639)	**0.017**
PWMH, %ICV	−4.856 (−6.433–3.279)	< –**0.001**	PWMH, %ICV	−2.720 (−5.299–0.141)	**0.044**	PWMH, %ICV	−2.679 (−4.909–0.450)	**0.022**
DWMH. %ICV	−1.185 (−2.933–0.563)	0.190	DWMH. %ICV	−0.796 (−3.097–1.506)	0.501	DWMH. %ICV	−1.248 (−3.241–0.745)	0.225
*Global cognition*								
MMSE	1.576 (−0.749–3.902)	0.192	MMSE	1.986 (−0.672–4.643)	0.151	MMSE	0.332 (−2.538–3.203)	0.822
MoCA	2.544 (0.304–4.784)	**0.033**	MoCA	1.913 (−0.433–4.260)	0.120	MoCA	1.560 (−1.462–4.583)	0.319

*Note:* Data were adjusted for age, sex, hypertension, diabetes mellitus, and educational years. Bold values indicate the significance of *p* values.

### 3.4 OCTA‐Derived Measures Are Associated With Increased SVD and PWMH Volumes in DLB

Elevated SVD burden and PWMH volumes in DLB were significantly associated with reduced SVC, DVC, and CC perfusions, respectively (all *p* < 0.05). No significant association was observed between DWMH volumes and OCTA‐derived measures in DLB (all *p* > 0.05).

Further, we found that lower MoCA scores in DLB were significantly associated with lower SVC density (ß = 2.544, 95% CI 0.304 to 4.784, *p* = 0.033). No significant association was observed between MMSE scores and OCTA‐derived measures in DLB (*p* > 0.05).

### 3.5 Mediation Analyses Between OCTA‐Derived Measures and Global Cognitive Measures in DLB

**FIGURE 2:**
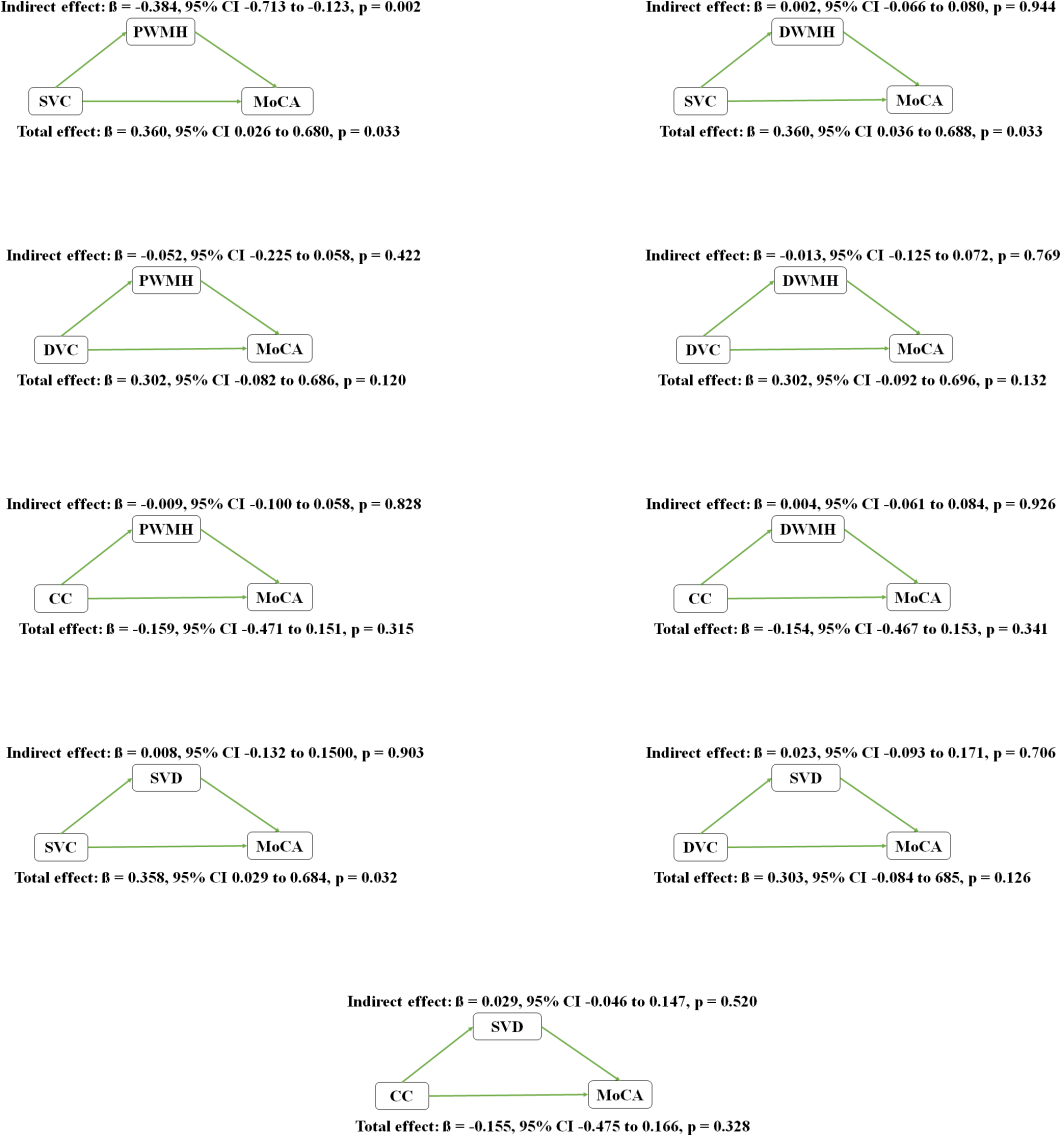
Mediation analyses between OCTA‐derived measures and global cognitive measures in DLB. The association between lower SVC density and lower MoCA scores in DLB was partially mediated by elevated PWMH volume.

The mediation analyses between OCTA‐derived measures and global cognitive measures in DLB are shown in Figure 2. We found that the association between lower SVC density and lower MoCA scores in DLB was partially mediated by elevated PWMH volume (ß = −0.384, 95% CI −0.713 −0.123, *p* = 0.002).

## 4 Discussion

In this study, we demonstrate that retinal microvascular impairment, quantified using OCTA, may be a prominent feature of DLB and is closely associated with Alzheimer's‐related plasma biomarkers, SVD, and cognitive decline. By integrating retinal imaging, blood‐based biomarkers, and neuroimaging measures, our findings provide converging evidence that neurodegenerative and vascular pathologies in DLB may be tightly interconnected and can be captured through a non‐invasive retinal assessment.

Individuals with DLB exhibited marked reductions in vessel density across the SVC, DVC, and CC compared with CU. Among these metrics, CC density demonstrated the highest discriminative performance, suggesting that choroidal microvascular compromise may be particularly sensitive to disease‐related pathology in DLB. These findings are consistent with emerging reports of retinal and choroidal microvascular alterations in DLB and extend prior work in ad [10, 11, 31] and PD [13], supporting the concept that retinal vascular dysfunction may be a shared feature across neurodegenerative disorders. The retina and brain share common embryological origins, microvascular architecture, and blood–tissue barrier properties. Consequently, retinal microvascular alterations may reflect systemic or cerebral microangiopathy rather than isolated ocular pathology. In DLB, where α‐synuclein aggregation frequently coexists with amyloid‐*β* and tau pathology [32], retinal vascular compromise may arise from a convergence of neurodegenerative, inflammatory, and vascular mechanisms. Our findings suggest that OCTA‐derived metrics may be sensitive to these pathological processes and may capture disease‐relevant vascular alterations in vivo.

A key finding of this study is the robust association between retinal microvascular impairment and plasma biomarkers of Alzheimer's‐related pathology. Reduced retinal vessel densities were associated with lower plasma A*β*42 and A*β*42/40, as well as elevated p‐tau217. Notably, the combined p‐tau217/A*β*42 showed consistent associations across all retinal vascular layers, highlighting its potential value as an integrated marker of amyloid and tau pathology in DLB. These observations align with growing evidence that Alzheimer's‐related pathology is common in DLB and may contribute to the clinical heterogeneity and disease severity [33, 34]. Amyloid deposition and tau phosphorylation have been linked to vascular dysfunction through endothelial injury, impaired neurovascular coupling, and blood–brain barrier disruption [35]. Our findings suggest that similar mechanisms may extend to the retinal microvasculature, supporting the notion that retinal OCTA metrics may reflect molecular pathology traditionally assessed through cerebrospinal fluid or positron emission tomography.

SVD and WMHs were significantly more severe in individuals with DLB than in CU, consistent with prior neuroimaging studies [36, 37, 38]. Importantly, retinal microvascular impairment was associated with greater SVD burden and PWMH volume, but not deep WMH volume. This spatial specificity is notable, as PWMHs are thought to be more closely related to hypoperfusion and impaired venous drainage, whereas deep WMHs may reflect heterogeneous pathological processes [39]. The observed associations support the concept that retinal microvascular changes may serve as a peripheral marker of cerebral microangiopathy in DLB. Given the shared hemodynamic and structural features between retinal and cerebral small vessels, OCTA imaging may offer a practical and scalable approach to assessing vascular contributions in DLB, particularly in settings where advanced neuroimaging is not readily available.

We found that reduced SVC density was associated with worse global cognitive performance, as measured by MoCA. Mediation analyses further indicated that this association was partially mediated by PWMH volume, suggesting that retinal microvascular impairment may reflect upstream vascular pathology that contributes to white matter injury and cognitive decline. These findings reinforce the importance of vascular mechanisms in DLB‐related cognitive dysfunction and support a model in which microvascular compromise contributes to white matter damage, thereby exacerbating cognitive impairment [40, 41, 42]. The lack of a significant association with MMSE scores likely reflects the limited sensitivity of this instrument to executive and attentional deficits, which are prominent in DLB.

APOE ε4 carriers in DLB exhibited more pronounced retinal microvascular impairment, higher SVD burden, and elevated p‐tau217 levels compared with non‐carriers. This finding is consistent with the established role of APOE ε4 in promoting amyloid accumulation, tau pathology, and vascular dysfunction [11, 12, 19]. In the context of DLB, APOE ε4 may amplify both neurodegenerative and vascular pathways, leading to more severe microvascular compromise in both the brain and retina. Our results suggest that genetic susceptibility may influence the extent of retinal and cerebral vascular pathology in DLB and underscore the importance of considering APOE genotype when interpreting retinal biomarkers.

Together, these findings support OCTA as a promising non‐invasive imaging tool that captures convergent vascular and neurodegenerative pathology in DLB. Retinal imaging is rapid, widely accessible, and cost‐effective, making it well‐suited for large‐scale screening, disease monitoring, and integration into multimodal biomarker frameworks alongside plasma biomarkers and neuroimaging. From a research perspective, OCTA imaging may facilitate the study of neurovascular mechanisms in DLB and aid in stratifying patients based on vascular and molecular burden. Clinically, it holds potential for identifying individuals at higher risk of rapid cognitive decline or mixed pathology.

Several limitations should be considered when interpreting our findings. First, the cross‐sectional design precludes inference regarding temporal or causal relationships among retinal microvascular alterations, plasma biomarkers, SVD, and cognitive impairment. Although mediation analyses were performed, these represent statistical modeling of associations and do not provide mechanistic or directional evidence. Longitudinal studies are required to determine whether retinal microvascular changes precede, parallel, or follow cerebral vascular and neurodegenerative processes in DLB. Second, the sample size was modest, particularly for subgroup analyses stratified by APOE ε4 carrier status. The relatively small number of ε4 carriers limits statistical power and increases susceptibility to sampling variability. Although regression models were adjusted for age and other covariates, residual imbalance between subgroups cannot be fully excluded. Replication in larger, multi‐center cohorts is warranted. Third, participants were recruited from a single tertiary medical center, which may limit generalizability to broader or community‐based DLB populations. In addition, all cases met clinical diagnostic criteria for probable DLB, but neuropathological confirmation was not available. As mixed pathology is common in DLB, the extent to which underlying AD co‐pathology contributed to the observed associations cannot be definitively established. Fourth, although major vascular risk factors were adjusted for in multivariable models, residual confounding by unmeasured systemic or microvascular factors remains possible. Retinal microvascular measures may be influenced by systemic hemodynamic or metabolic variables that were not comprehensively assessed. Fifth, plasma samples were collected in the morning, but not uniformly under fasting conditions. While current evidence suggests that plasma amyloid‐*β* and phosphorylated tau concentrations are relatively stable with respect to fasting status, potential variability related to pre‐analytical conditions cannot be entirely excluded.

In summary, retinal microvascular impairment in DLB is closely associated with Alzheimer's‐related plasma biomarkers, cerebral small vessel disease, and cognitive dysfunction. These findings highlight the retina as a valuable window into the complex interplay between neurodegeneration and vascular pathology in DLB and support the integration of retinal OCTA into multimodal biomarker strategies for this disorder.

## Author Contributions

Study concept and design: W.R.K., Z.W., X.L., Q.T., Data Acquisition: X.L., Z.W., Q.T., S.H., Y.H., Y.G., C.J., M.Y., W.R.K. Data Analysis and Interpretation: W.R.K., Z.W., Q.T., X.L. Drafting of the manuscript: W.R.K., Z.W., Q.T., X.L. Critical review of the manuscript: W.R.K., Z.W., X.L. All authors reviewed and approved this version of the manuscript.

## Funding

The project is supported by the Headache Specialty Care Program at the First Affiliated Hospital of Wenzhou Medical University (009).

## Ethics Statement

The protocol of this study was approved by the Ethics Committee of The First Affiliated Hospital of Wenzhou Medical University (Ethics Number: KY2021‐153). All participants provided written informed consent before enrolling in the study.

## Consent

All authors have read the manuscript and agree to its publication. Table S1 displays the ROC analysis of the OCTA‐derived measures in differentiating DLB from CU. CC showed the highest AUC in differentiating DLB from CU.

## Conflicts of Interest

The authors declare that they have no conflicts of interest. The sponsor had no role in the study design, data collection, or writing of the manuscript.

## Supporting information


**Table S1:** ROC analysis of the OCTA‐derived measures in differentiating DLB from CU.

## Data Availability

The data that support the findings of this study are available on request from the corresponding author. The data are not publicly available due to privacy or ethical restrictions.
