# Supplementary material for: Retinal Microvascular Dysfunction Reflects Vascular and Alzheimer's‐Related Pathology in Dementia With Lewy Bodies
Source: CNS Neurosci Ther. 2026 Apr 21;32(4):e70891. doi: 10.1002/cns.70891 (PMC13098535; doi:10.1002/cns.70891)
Supplement: Supplementary file 1 — Table S1: ROC analysis of the OCTA‐derived measures in differentiating DLB from CU. [file CNS-32-e70891-s001.docx]

**Supplementary Table 1. ROC analysis of the OCTA-derived measures in differentiating DLB from CU.**

| OCTA derived measures | AUC | Youden Index | Sensitivity | Specificity |
| --- | --- | --- | --- | --- |
| SVC, % | 0.816 | 0.587 | 0.684 | 0.903 |
| DVC, % | 0.797 | 0.518 | 0.825 | 0.694 |
| CC, % | 0.913 | 0.830 | 0.895 | 0.935 |

OCTA, optical coherence tomography angiography; AUC, area under the curve; SVC, superficial vascular complex; DVC, deep vascular complex; CC, choriocapillaris
